# In vitro photodynamic therapy of methylene blue-loaded acetyl resistant starch nanoparticles

**DOI:** 10.1186/s40824-022-00273-7

**Published:** 2022-06-27

**Authors:** In-Kyu Park, Do-Bin Ju, Amal Babu, Jeong-Cheol Lee, Young Jin Pyung, Chong-Su Cho, Hyun-Joong Kim

**Affiliations:** 1 Department of Biomedical Sciences, BK21 PLUS Center for Creative Biomedical Scientists, Chonman National University Medical School, Gwangju, 61469 South Korea; 2 Department of Agricultural Biotechnology, Seoul National University, Seoul, 08824 South Korea; 3 Research Institute of Agriculture and Life Sciences, Seoul National University, Seoul, 08824 South Korea; 4 Program in Environmental Materials Science, Department of Agriculture, Forestry and Bioresources, Seoul National University, Seoul, 08824 South Korea

**Keywords:** Polymeric nanoparticles, Methylene blue, Resistant starch, Photodynamic therapy

## Abstract

**Background:**

Combination therapies comprising multiple methods, such as photodynamic therapy have been applied to be complements chemotherapy as they increase the therapeutic efficiency by enabling the intelligent drug delivery to target sites by exposing the photosensitizer to light and activating it in the tumor tissue. This study evaluated in vitro photodynamic therapy of methylene blue (MB)-loaded acetyl resistant starch (ARS) nanoparticles (NPs).

**Methods:**

ARS was synthesized by the reaction between resistant starch (RS) and acetic anhydride. MB-loaded ARS NPs and ARS NPs were prepared by a single emulsion method. Synthesized ARS was measured by NMR. Prepared ARS NPs and MB-loaded ARS NPs were characterized by transmission electron microscopy (TEM), dynamic light scattering (DLS), X-ray diffraction, UV/Vis, and circular dichroism (CD). MB-loaded ARS NPs were treated in mouse colon cancer cells (CT-26) and they were treated under near-infrared (NIR) laser irradiation.

**Results:**

Synthesis of ARS was confirmed by NMR and the degree of substitutions in the ARS was 7.1. The morphologies of ARS NPs observed by TEM were spherical shapes and the particle sizes of ARS NPs were 173.4 nm with a surface charge of − 17.24 mV. The d-spacing of ARS NPs was smaller than those of RS and the conformational changes of RS occurred by the formation of self-assembled polymeric NPs with induction of CD of the MB by chiral ARS NPs. The phototoxicity of CT-26 cells treated by MB-loaded ARS NPs dramatically decreased in a dose-dependent manner under NIR laser irradiation compared to free MB.

**Conclusion:**

This study demonstrated the ordered nanosized structures in the ARS NPs and conformational change from random coil structure of RS to alpha-helices one of ARS occurred and CD of the achiral MB was induced. The MB-loaded ARS NPs showed a higher generation of reactive oxygen species (ROS) in the CT-26 cells than free MB with the NIR laser irradiation and resulting in phototoxicity under irradiation.

**Supplementary Information:**

The online version contains supplementary material available at 10.1186/s40824-022-00273-7.

## Introduction

Polymeric nanoparticles (NPs) have been widely used for chemotherapy because they can be designed to control diverse sizes, tunable physicochemical properties, and versatile topologies [1–4]. Also, they can be used to deliver hydrophobic cancer drugs into the cells due to their easy internalization of them with the cellular membrane through the endocytosis mechanism [5], although they still have disadvantages such as instability under physiological conditions for long periods, side effects, drug delivery efficiency, and inability to release the drug at specific pathologic sites [6].

Recently, combination therapies comprising multiple methods, such as photodynamic therapy (PDT) have been tried to be complements to chemotherapy because they can increase the therapeutic efficiency by enabling the intelligent drug delivery to target sites and fewer side effects [7–9]. The PDT currently used in the clinic as an adjunctive therapy has been attractive in the treatment of various cancer because it is possible to expose the photosensitizer to light and activate it in the tumor tissue [10] although the photosensitizer (PS) should satisfy several points, such as purity, tumor selectivity, minimal toxicity, rapid systemic clearance and activation at a long wavelength with high photochemical reactivity [11]. Among PS, methylene blue (MB) approved by the FDA for use in methemoglobinemia has been used for the PDT because the MB provides an excellent penetration in the cell membrane with a small concentration in the mitochondria, lysosome, and DNA due to its benzene ring, and good quantum yield with low cost-effectiveness [12] although clinical use of MB for PDT has been limited due to the low therapeutic efficacy following systemic administration [10]. To overcome the above-mentioned limitation, the MB was loaded into the nanocarriers [13].

Resistant starch (RS) as the mixture of amylose and amylopectin was reported that escaped in the proximal gut and was fermented in the ileum and colon by gut microbiota [14] similar to fibers. Supplementation with RS in humans showed increased fecal short-chain fatty acids (SCFAs) [15] and the supplementation with the RS in rodents improved inflammatory status [16].

There has been growing interest in the aggregation of dyes as the guest onto optical active polymer templates as the host due to the noncovalent binding and then unique photophysical properties, although covalent linking between guest and host was reported [17, 18]. Interestingly, the induction of circular dichroism (CD) is termed as induced circular dichroism of the guest molecules, such as bilirubin [19], dye [20], and diazirin chromophore [21] through the interaction of optical active host molecules, such as polypeptide [22], polysaccharide [16], and DNA [23] occurred in the UV or visible region of the guest molecules.

In this study, we want to report the structural properties of acetyl RS (ARS) NPs and MB-loaded ARS NPs. Also, it is particularly interesting to study in vitro PDT of MB-loaded ARS NPs.

## Methods

### Materials

RS (mixture of 40 wt.-% of amylopectin and 60wt.- % of amylose) was provided by Ingredion (Westchester, IL, USA). The other chemicals were provided by Sigma-Aldrich (St. Louis, MO, USA).

### Synthesis of acetyl RS (ARS) and preparation of ARS NPs

At first, ARS was synthesized by the previously described similar method [24]. One gram of RS was dissolved in 10 ml of dimethyl sulfoxide (DMSO) at 54 °C, and 0.1 mol% dimethyl aminopyridine (DMAP) per RS sugar residues as a catalyst and 10 ml of acetyl anhydride were added to the above solution. The reaction was done at 54 °C for 48 h with nitrogen conditions. After the reaction, the above reactant was firstly dialyzed in DMSO to remove unreacted acetic acid and dialyzed in distilled water, and the obtained precipitates were freeze-dried.

### Preparation of ARS NPs

ARS NPs were prepared by a single emulsion method. Briefly, 100 mg of ARS was dissolved in 5 ml of dichloromethane (DCM) to prepare the organic solution. The ARS solution was added dropwise into 10 ml of 2% (w/v) poly (vinyl alcohol) (PVA) and the mixture was sonicated with the Ultrasonic Processor (Sonics Vibra Cell™, Newtown, Australia) at 60 amplitude, 4 min, and 10-sec pulser. The emulsion was firstly dropped into 40 ml of 1% (w/v) PVA solution and then stirred with an IKA RW20 mechanical stirrer at 600 rpm for 4 h at room temperature to evaporate the DCM. The NPs thus formed were collected with ultracentrifugation at 14,000 rpm for 10 min and rinsed with distilled water three times.

### Confirmation of ARS synthesis and characterization of ARS NPs

ARS synthesis and content of acetyl groups in the ARS were confirmed by 600 MHz ^1^H-nuclear magnetic resonance (NMR) spectroscopy (AVANCE 600, Bruker, Germany). The morphologies of ARS NPs were observed by transmission electron microscopy (TEM) (Talos L120C, FEI, Czech). The sizes of ARS NPs were measured by a dynamic light scattering (DLS) spectrophotometer (DLS-7000, Otsuka Electronics, Osaka, Japan). The zeta potentials of ARS NPs were measured by electron light scattering (ELS) spectrophotometer (DLS-7000, Otsuka Electronics, Osaka, Japan).

### Preparation of MB-loaded ARSNPs

MB-loaded ARS NPs were also prepared by a single emulsion method. Briefly, the mixture of 5 mg of MB dissolved in 1 ml of ethanol (EtOH) and 100 mg of ARS dissolved in 5 ml of DCM was obtained to prepare the organic solution. The mixture solution was added dropwise into 10 ml of 2% PVA and sonicated with an Ultrasonic Processor at 60 amplitude, 4 min, and 60-sec pulser. The emulsion firstly dropped into 40 ml of 1% PVA solution was stirred with an IKA RW20 mechanical stirrer at 600 rpm for 4 h at room temperature to evaporate DCM/EtOH. The NPs thus formed were collected with ultracentrifugation at 14,000 rpm for 10 min and rinsed with distilled water three times.

### Determination of loading content of MB in the MB-loaded ARS NPs

To determine the amount of MB loaded into the ARS NPs was determined by the UV-VIS absorbance of MB. Briefly, a stock solution of MB (1 mg/ml) was dissolved in 1 ml of distilled water and further diluted to obtain different concentrations via the dilution process (15.62, 7.81, 3.90, 1.95, 0.97, 0.48, 0.24, 0.12, and 0.061 μg mL^− 1^) of MB and analyzed at 664 nm and plotted the standard calibration curve.

### Wide-angle X-ray diffraction (WAXRD) and small-angle X-ray spectrometer (SAXS) measurements

WAXRD was measured by a D8 ADVANCE diffractometer (Bruker DAVINCI, Germany) equipped with a Cu radiation source with a wavelength (*λ*) of 1.5418 Å. WAXS diffractograms were obtained by measuring at 40 kV. 40 mA, and at a scan speed of 0.5 °/sec in the range of 0–40 ° 2*θ* range, where *θ* is the angle of incidence of the X-ray beam at room temperature. SAXS spectroscopy was obtained using a 1.54189 Å synchrotron X-ray beam (System model: Small Angle X-ray Scattering Spectrometer, Xenocs, France). The distance of sample-to-detector distance was 300 nm and the SAXS data was measured for 600 s under ambient conditions.

### Measurement of circular dichroism (CD) spectroscopy

The UV/Vis spectra were measured by a UV/Vis spectrometer (Cary 100, Agilent, USA) at room temperature. The CD spectrum was measured with Chirascan plus (Applied Photophysics, UK) at room temperature by a quartz cell with an optical path length of 1 mm. CD spectra were accumulated three times of 1 nm with a scan speed of 50 mm/min. The induced CD of MB-loaded ARS NPs was obtained as the CD of MB-loaded ARS NPs minus the CD of MB and ARS NPs measured at the same wavelength and expressed as ellipticity in millidegrees [25].

### 1,4 Diphenyl-2,3-benzofuran (DPBF) assay for singlet oxygen (^1^O_2_) generation test

The ability of MB-loaded ARS NPs and free MB to generate ^1^O_2_ was tested using DPBF. MB-loaded ARS NPs rapidly mixed with DPBF (300 μM) were put in an optical path length of 1 cm cuvette. Subsequently, the samples were irradiated by a NIR laser of 671 nm at a power density of 100 mW/cm^2^. Every one-minute corresponding absorbance at 470 nm was recorded using a UV-vis-NIR spectrophotometer.

### Cell culture

The mouse colon cancer (CT-26) cells were grown in Dulbecco’s Modified Eagle Medium (DMEM) with 10% fetal bovine serum and 1% antibiotic at 37 °C in a humidified incubator with 5% CO_2_ [26].

### Colorimetric WST-1 cellular viability assay

CT-26 cells were seeded overnight at a density of 1 × 10^4^ cells per well in a 96-well plate. Subsequently, the culture medium was aspirated, and the cells were treated with varying concentrations of MB (0, 20, 10, 5, 2.5, 1.25, 0.62, 0.31 μg mL^− 1^) in DMEM for 24 h incubation at 37 °C. To determine in vitro phototoxicity, CT-26 cells were seeded into a 96-well plate (1 × 10^4^ cells/well). MB-loaded ARS NPs and free MB were added to these cells and incubated for 6 h, replacing the culture media with fresh media, and then followed by 5 min of laser irradiation (671 nm laser, 100 mW/cm^2^) into 96-well plates. Then the cells were then incubated with WST-1 solution (10 μL) for 1 h and absorbance was measured at 440 nm.

### Detection of intracellular ROS: DCFH-DA staining

The CT-26 cells were seeded into a 12-well plate with a density of 1 × 10^5^ cells/ well and cultured in a humidified atmosphere containing 5% CO_2_ at 37 °C for 24 h. Cells were further incubated with free MB, MB-loaded ARS NPs (at MB equivalent dose of 10 μg mL^− 1^) for 6 h and replace the medium with fresh medium and irradiate using a laser with a power of 671 nm laser, 100 mW/cm^2^. After irradiation incubates with 25 μM DCFH-DA for 20 min. The fluorescence microscope was used to obtain the images.

### Statistical analysis

The results are presented as the means±standard deviation for results obtained from three independent trials.

## Results

### Synthesis of ARS and characterization of ARS NPs

**Fig. 1:**
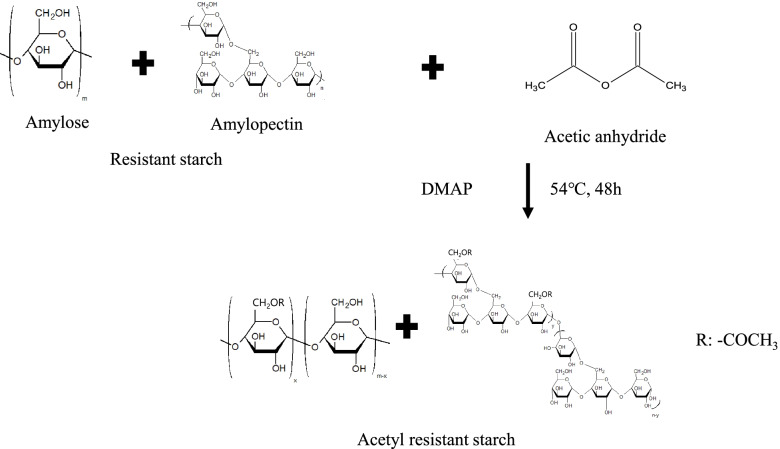
Chemical reaction scheme of ARS

The reaction scheme of ARS synthesis is shown in Fig. 1. The synthesis of ARS was confirmed by ^1^H-NMR measurement as shown in suppl. Fig. 1. It was obtained that peaks of 1.9–2.1 ppm were assigned to the protons of methyl, and peaks of 4.58–5.50 ppm were assigned to the protons of sugar, which belongs to the C_1_ position of α-1,6 and α-1,4 glycosidic bonds, respectively, appeared in the NMR spectrum of ARS. The degree of substitutions (DS) of acetyl groups in the ARS was obtained by using the equation described by Elomaa et al. [27]. It was found that the DS in the ARS was 7.1.

**Fig. 2:**
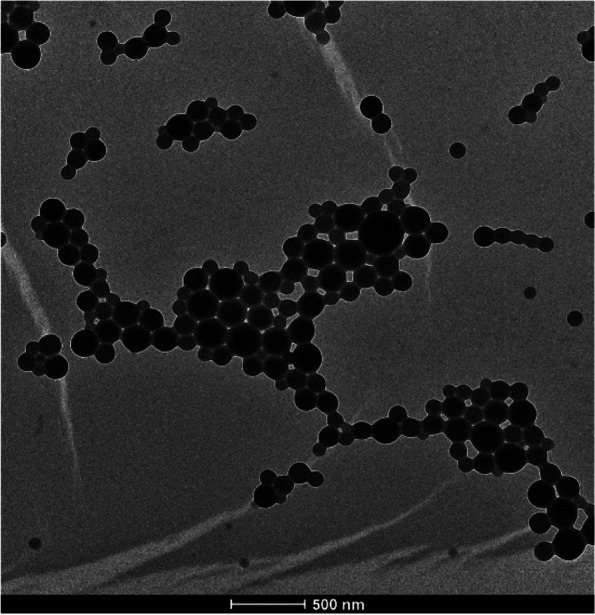
Morphologies of ARS NPs observed by TEM

**Fig. 3:**
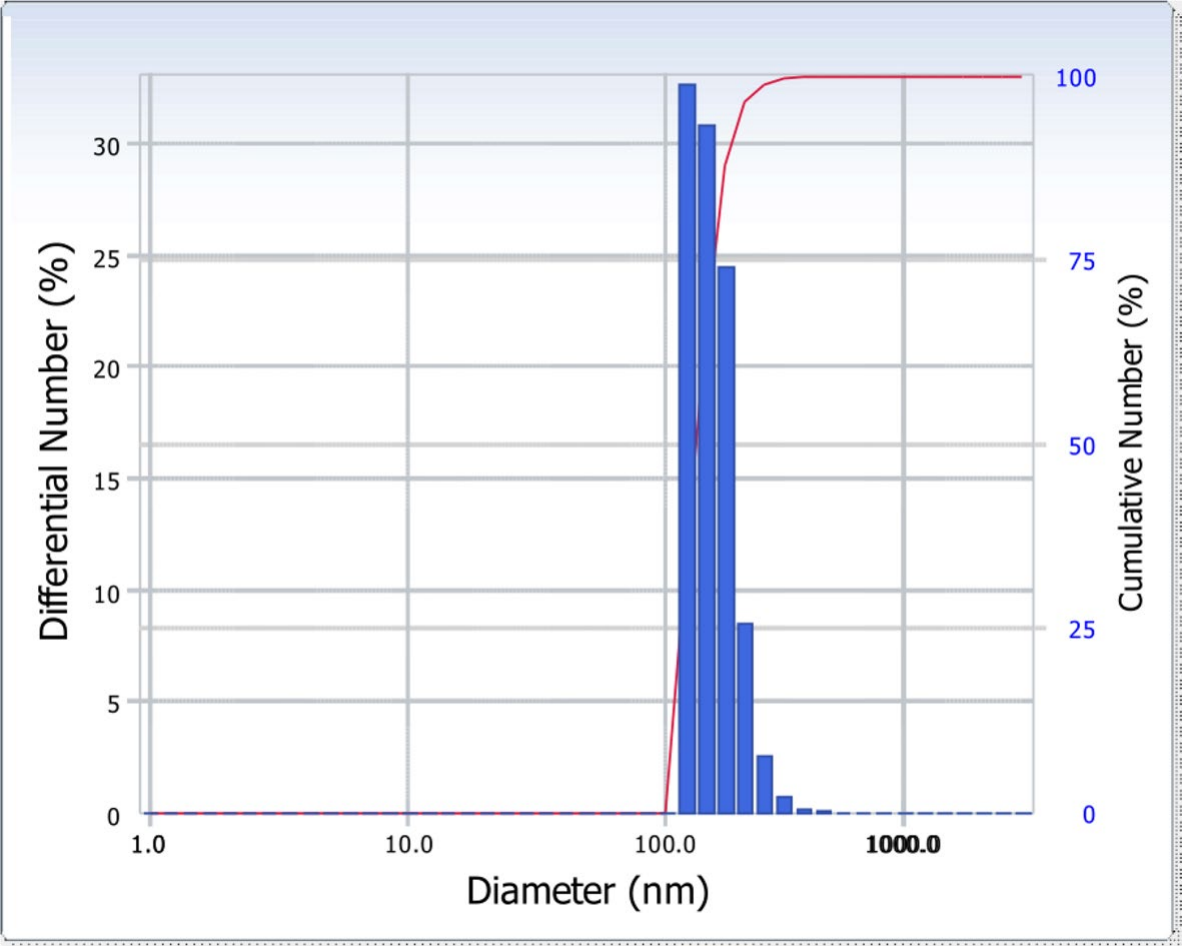
Particle sizes of ARS NPs measured by DLS

**Fig. 4:**
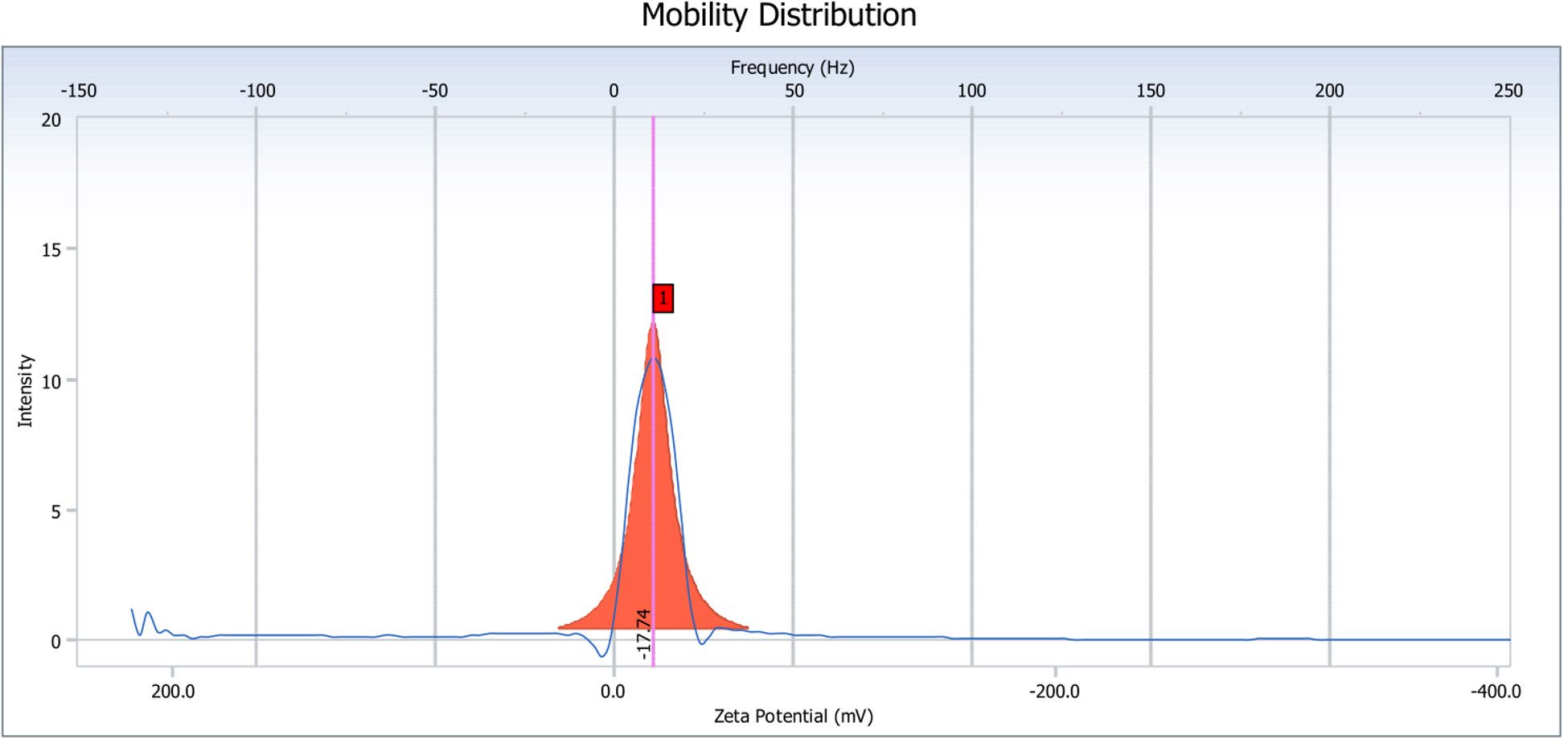
Zeta potential of ARS NPs measured by ELS

The morphologies of ARS NPs prepared by the single emulsion method after conjugation of hydrophobic acetyl groups to hydrophilic hydroxyl ones in RS were observed as spherical shapes with the sizes between 100 nm and 200 nm as shown in Fig. 2 owing to the dry state although the fusion of ARS NPs was observed due to the retaining the bound water to form hydrogen bonds in the ARS NPs whereas the particle sizes of ARS NPs measured by results of DLS were 173.4 nm with polydispersity index of 0.096 as shown in Fig. 3. Also, the surface charge of the ARS NPs measured by ELS was − 17.24 mV as shown in Fig. 4, the indication of negative zeta potential owing to the unreacted carboxyl groups in the ARS NPs.

### Measurement of WAXRD and SAXS

**Fig. 5:**
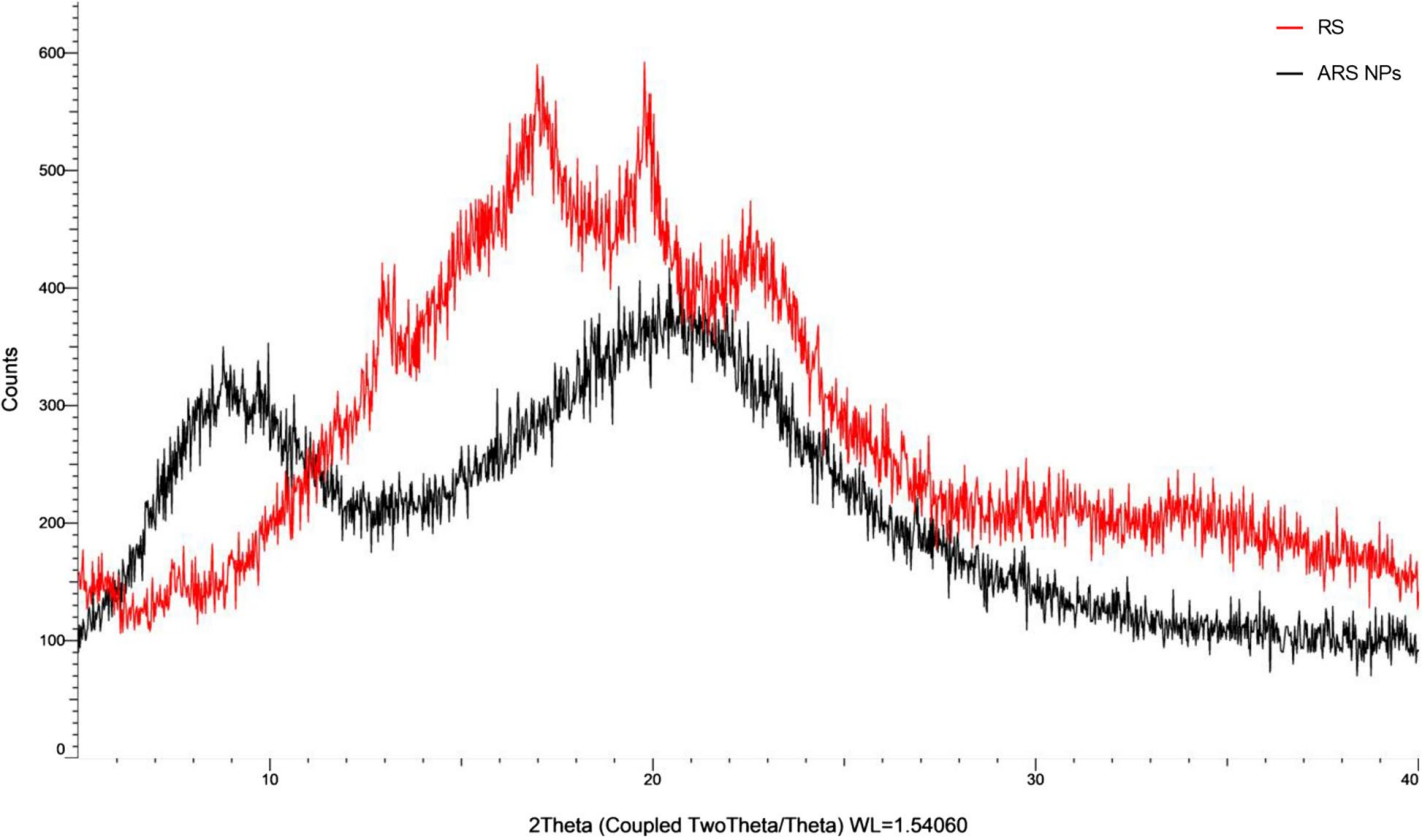
XRD patterns of ARS NPs and RS

**Fig. 6:**
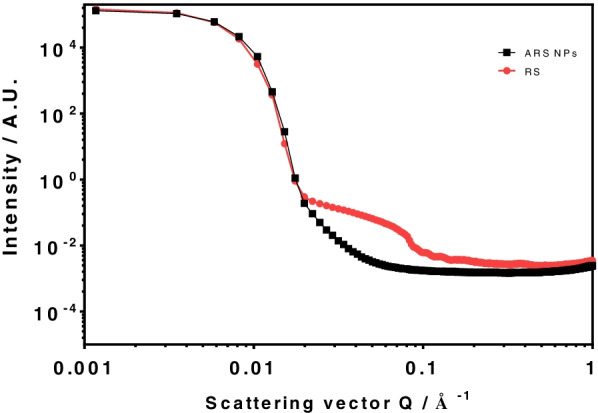
SAXS diffraction pattern of ARS NPs

XRD patterns for RS and ARS NPs are shown in Fig. 5. As shown in Fig., the XRD pattern of RS exhibits strong diffraction peaks at 2 *θ* values of about 13.0 °, 17,0 °, 19.9 °, and 22.8 ° that correspond to d-spacing of 0.68, 0.52, 0.45, and 3.91 nm, respectively. On the other hand, the XRD patterns of ARS NPs exhibit strong reflections peaks at 2 *θ* values of about 8.9 ° and 20.5 ° that corresponding to d-spacing of 0.47 and 1.07 nm, respectively. It was found that d-spacings of ARS NPs were smaller than those of RS and four sharp peaks of RS became less sharp than those of RS. SAXS diffraction pattern for RS and ARS NPs is shown in Fig. 6. As shown in Fig., a SAXS diffraction slope of ARS NPs was less sharp than AR, which is similar results of XRD patterns.

### Measurement of UV and CD

**Fig. 7:**
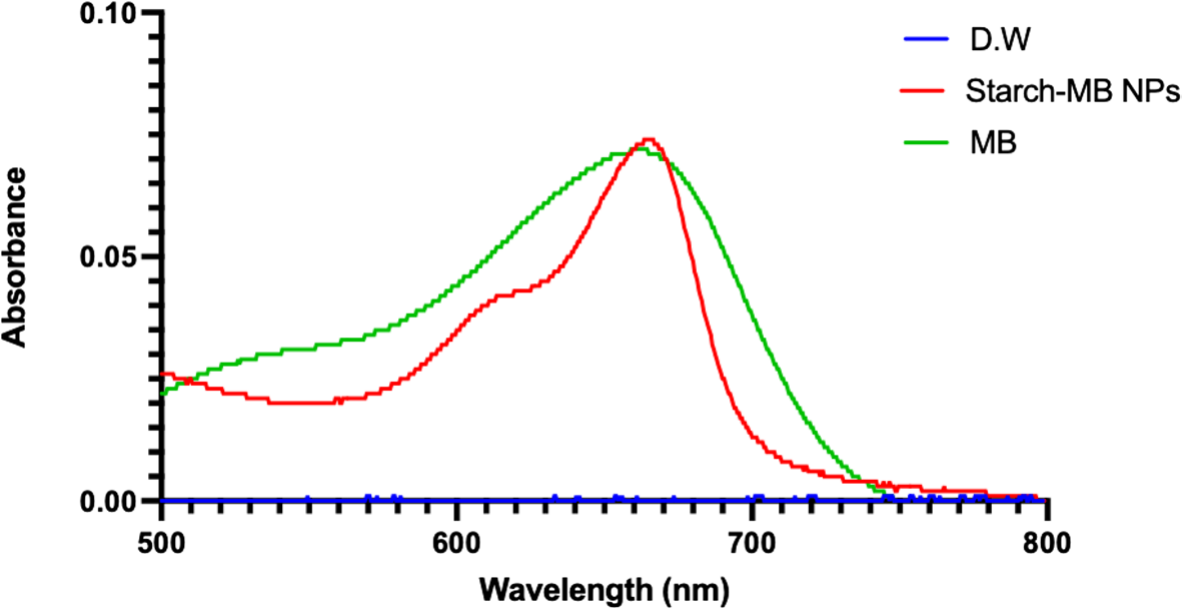
UV-vis absorption spectra of MB and MB-loaded ARS NPs in water at room temperature

**Fig. 8:**
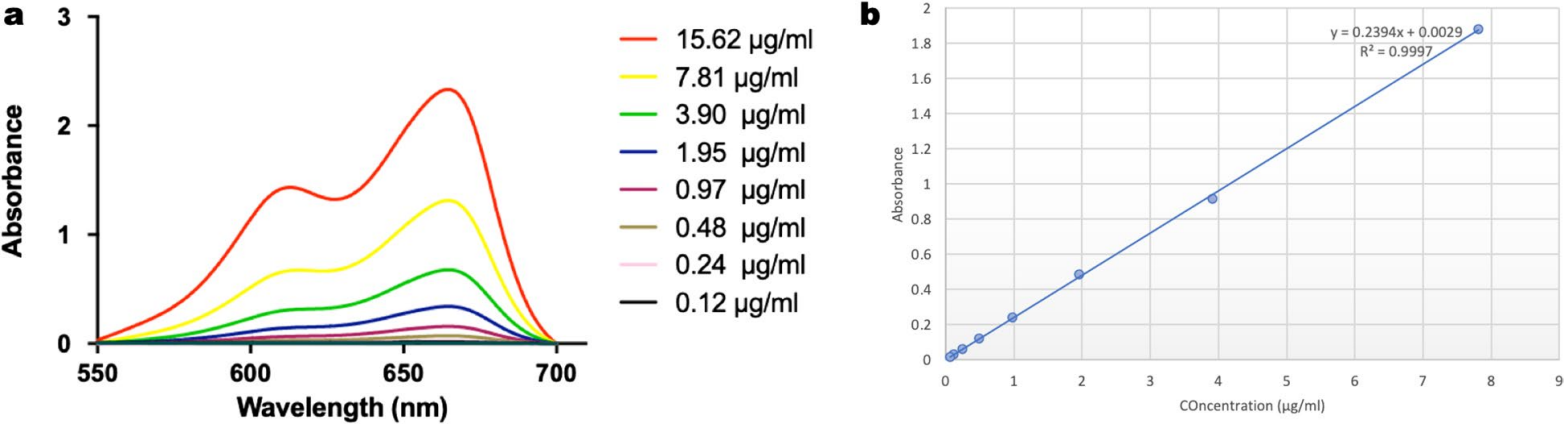
**A** UV-VIS spectra of MB solutions at various concentrations: **B** standard calibration curve of MB solution at various concentrations

Figure 7 shows the absorption spectra of free MB, MB-loaded ARS NPs. The characteristic absorption peaks for free MB are at λ max 664 nm due to the monomeric state with the shoulder at 610 nm due to the aggregated state whereas the MB-loaded ARS NPs absorption peaks are observed at 668 due to the monomeric state and 610 nm due to the aggregated state. The loading capacity of MB in MB-loaded ARS NPs was estimated using the standard calibration curve. The calibration curve of MB obtained at 664 nm as shown in Fig. 8B was used for determining the concentration of MB in the MB-loaded ARS NPs. A plot of concentration versus absorbance value as shown in Fig. 8A is linearly dependent on MB concentration. The accuracy was excellent with a coefficient correlation (R^2^) of 0.997. The loading capacity of MB in the MB-loaded ARS NPs was estimated at 1.82 ± 0. 01 wt.-% using the calibration curve.

**Fig. 9:**
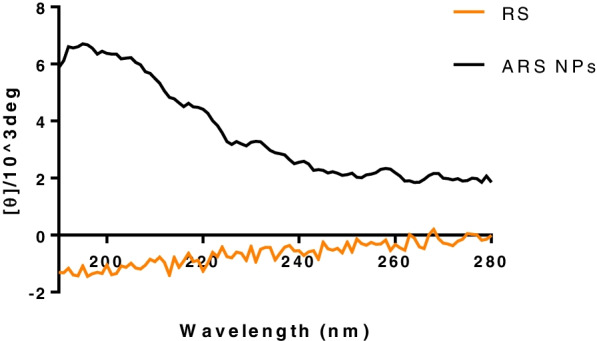
CD spectra of RS and ARS NPs in water at room temperature

Figure 9 shows CD spectra of RS itself and ARS NPs in water at room temperature. As shown in Fig., the RS exhibited a very weak negative absorption, the indication of the almost non-ordered structure. On the other hand, the ARS NPs exhibited two positive ellipticities at 208 and 230 nm for alpha-helices although 230 nm exhibited aggregated alpha-helices.

**Fig. 10:**
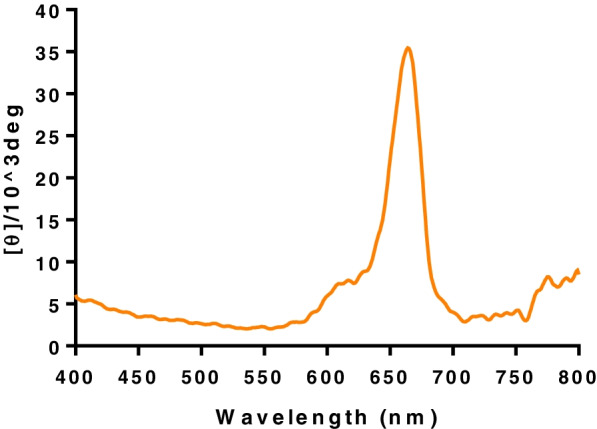
CD spectrum of MB-loaded ARS NPs in water at room temperature

Figure 10 shows the CD spectrum of the MB-loaded ARS NPs. The result indicated that strong and weak shoulder positive bands appeared in the CD spectrum at around the UV spectrum and the observed induced CD spectrum of the MB was matched with its UV absorption spectrum precisely.

**Fig. 11:**
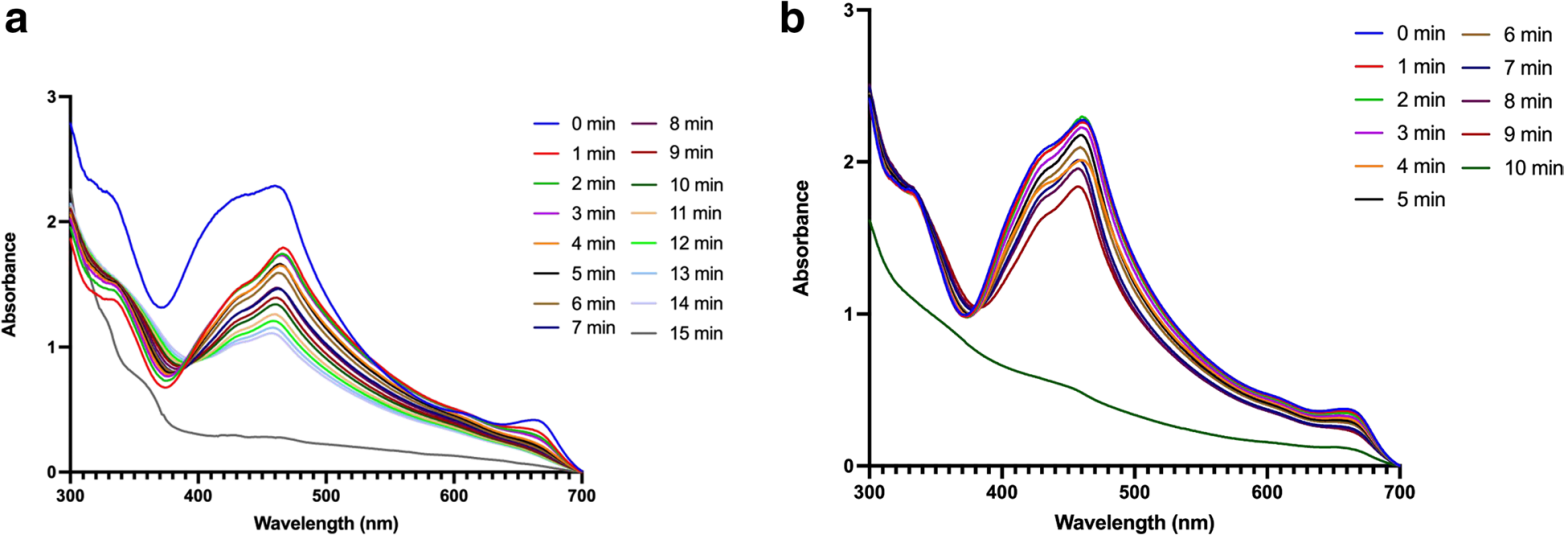
**A** UV-vis spectra of DPBF solution incubated with MB-loaded ARS NPs in distilled water: **B** UV-vis spectra of MB in distilled water under 671 nm irradiation against time

**Fig. 12:**
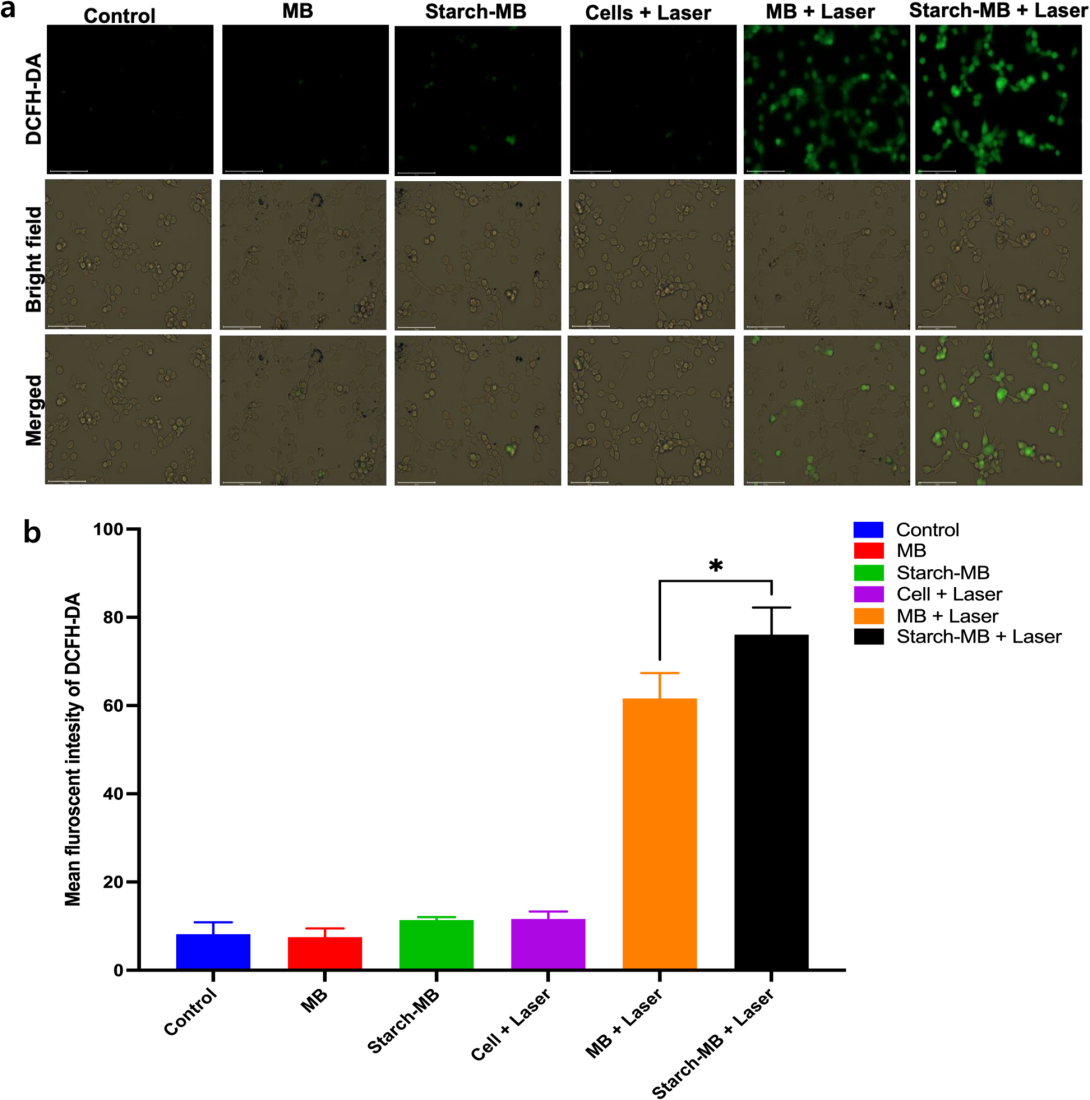
**A** Intracellular ROS generation of MB-loaded ARS NPs under light irradiation (671 nm, 100 mW/cm^2^, 5 min) was observed by fluorescence microscopy. Scale bar 100 nm. **B** Quantitative analysis of mean fluorescence intensity of DCFH-DA with image-j software. Data are presented as means ± SD. Statistical significance was calculated by student’s t-test (^*^P < 0.05) using GraphPad prism software Version 9.1.1

**Fig. 13:**
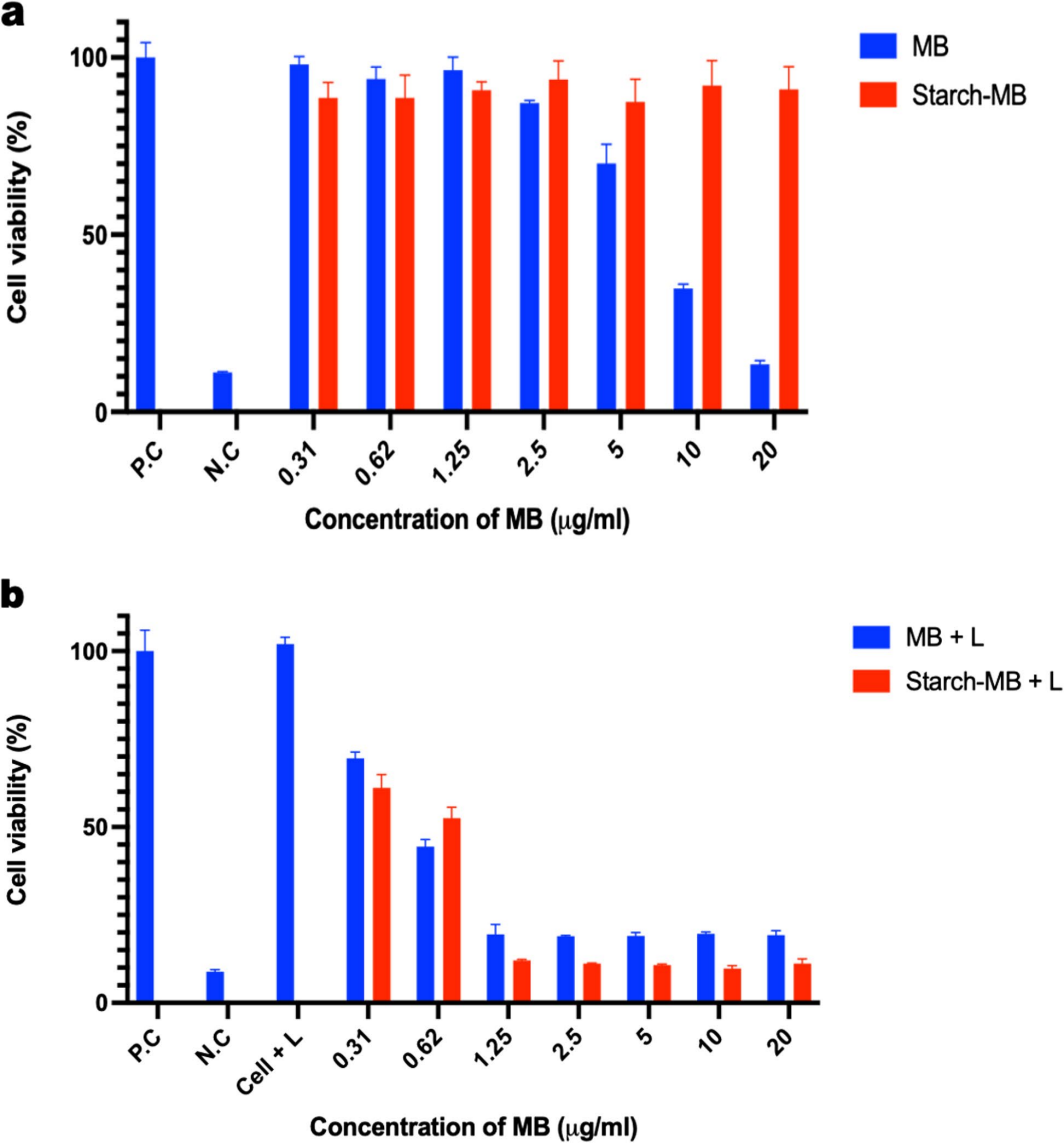
Cell viability of CT-26 cells treated with varying concentration of MB. **A** and **B** Cell viability of starch-MB and free MB with and without laser irradiation (671 nm, 100 mW/cm^2^)

To evaluate the photodynamic property of MB-loaded ARS NPs, DPBF was used for the detection of singlet oxygen (^1^O_2_), and the reaction was monitored by recording the absorption intensity of the DPBF at 470 nm against time. The decreased time of intensity of the absorption peak at 470 nm shows the amount of produced singlet oxygen as shown in Fig. 11. UV-vis spectra of MB-loaded ARS NPs (Fig. 11A) and free MB (Fig. 11B) under laser irradiation decreased significantly. Furthermore, intracellular ROS generation was studied in CT-26 cells by fluorescent microscopy. The common intracellular probe used to detect ROS is 2′,7′, dichlorofluorescein diacetate (DCFH-DA). As shown in Fig. 12 (A), the fluorescent microscope of DCF-DA was observed after the cells were irradiated with the NIR laser. The MB-loaded ARS NPs with laser irradiation showed high fluorescence signals as compared to the non-irradiated control group. (B) Quantitative analysis of mean fluorescence intensity of DCFH-DA with image-j software is shown in Fig. 12 (B). The weak ROS fluorescence was obtained from MB-loaded ARS NPs, MB, and control cells without irradiation group whereas MB-loaded ARS NPs showed significantly higher fluorescence intensity than non-irradiated groups. Hence, under NIR laser irradiation, MB-loaded ARS NPs can generate ROS in the intracellular environment. The results indicated that the MB-loaded ARS NPs generated ROS intracellularly in vitro. Also, the phototoxicity of MB-loaded ARS NPs was then evaluated by WST-1 assay. CT-26 cells were incubated with different concentrations of MB-loaded ARS NPs and free-MB. As shown in Fig. 13A, free MB shows toxicity with an increase in concentration. On the other hand, MB-loaded ARS NPs showed no evident cytotoxicity up to an MB concentration of 20 μg mL^− 1^. Furthermore, phototoxicity was also assessed by evaluating cell viability under 671 nm laser irradiation (100 mW/cm^2^) for 5 min with the same serial concentration of MB-loaded ARS NPs. As shown in Fig. 13B, the cell viability treated by MB-loaded ARS NPs decreased in a dose-dependent manner under laser irradiation.

## Discussion

In this study, we developed MB-loaded ARS NPs for in vitro photodynamic therapy because the photodynamic therapy can be used to complement chemotherapy as a combination therapy as it increases the therapeutic efficiency by exposing the photosensitizer to light and activating it in the tumor tissue. ARS NPs can be prepared by self-assembly of RS conjugated with acetic anhydride as hydrophobic groups and hydroxyl ones in AR through hydrophobic interactions. It is suggested that the reaction occurs between the primary hydroxyl groups of the AR and carboxylic acids of acetic anhydride after ring-opening through an esterification mechanism.

During the formation of polymeric NPs by self-assembly through hydrophobic interactions, it is suggested that conformational changes of ARS NPs occurred from random coil to alpha-helix in water by the formation of self-assembled polymeric NPs although the crystalline alignment of ARS NPs became less than RS owing to the introduction of the bulky hydrophobic acetyl groups into RS at solid-state. Also, the hydrophobic compound can be loaded into ARS NPs as the aggregation of MB due to the host-guest complexes and induction of CD of the MB occurred by chiral ARS NPs. It can be expected that the induction of CD of the MB as the guest molecules will have unique photophysical properties because chirality induction of the MB by the interaction of MB and ARS NPs can affect the photosensitivity of loaded MB in the ARS NPs although they should be checked in the future.

PS defined to induce photochemical reactions after irradiation with light have been recently used for biomedical applications. Therefore, they should be highly pure, stable, non-cytotoxic, have long-waved light for deep penetration into the target sites, fast excretion from healthy tissues, and be inexpensive. Among PS, MB as one of the second-generation PS approved by the FDA has been applied for basal cell carcinoma and methemoglobinemia because it can excellently penetrate a cell membrane with a small concentration [12]. However, the MB limits clinical use owing to low therapeutic efficacy following systemic administration and dark cytotoxicity in doses of MB over 20 μM [10]. Also, the MB does not show enough absorption between 700 and 1100 nm which is clinically ideal ptototherapeutic biological window for cancer cells because PS having those maximum wavelength can be deeply penetrated into cancer cells. Therefore, to improve these aforementioned shortcomings, the MB should be loaded into the nanocarriers. In this study, ARS NPs were used to load the MB because the RS escaped in the proximal gut and fermented in the ileum and colon due to the resistance of breakdown in small intestine and fermentation by the resident bacteria in the large intestine. It can be expected that MB-loaded ARS NPs will be promising in the treatment of colon cancer PDT.

The MB-loaded ARS NPs produced singlet oxygen similar to free MB under laser irradiation using DPBF for the detection of singlet oxygen because the DPBF reacted irreversibly with the singlet oxygen generated by the photo-excitation. As a result of intracellular ROS generation in CT-26 colon cancer cells by fluorescent microscopy using DCFH-DA as the intracellular ROS generator with laser irradiation, apparent green fluorescence was highly observed by MB-loaded ARS NPs than non-irradiated control group because the DCFH-DA enter the cells and is deacetylated to form the DCFH carboxylate anion. Under the presence of ROS in the cells, the DCFH reacts with the DCFH carboxylate anion and produces green fluorescence dichlorofluorescein without no evident cytotoxicity up to an MB concentration of 20 μg/ml for the MB-loaded ARS NPs whereas free MB shows toxicity with an increase of MB concentration, the suggestion of excellent biocompatibility of MB-loaded ARS NPs due to the encapsulation of MB in the ARS NPs.

## Conclusion

The ordered nanosized structures in the ARS NPs and conformational change from random coil structure of RS to alpha-helices one of ARS occurred by the formation of ARS NPs through the hydrophobic interaction of introduced acetyl groups in the ARS NPs. MB was loaded into the ARS NPs through ionic and hydrophobic bondings between MB and ARS NPs. The CD of the achiral MB was induced by the chiral microenvironment of ARS NPs. The MB-loaded ARS NPs showed a higher generation of ROS intracellularly in CT-26 cells than free MB with the NIR laser irradiation and resulted in the phototoxicity in a dose-dependent manner under irradiation, demonstrating a promising potential in photodynamic therapy. Especially, it will be expected to apply for the clinical treatment in colon cancer due to the specific characteristics of RS in the large intestine. However, photodynamic therapy of MB-loaded ARS NPs after designing of ARS NPs having enough maximum absorption above 700 nm to be deeply penetrated into cancer cells and having cancer cell targeting ligand to get selectivity for the targeted tumor sites without phototoxicity to normal tissues should be performed in vivo in the future.

## Supplementary Information

**Additional file 1: Supplementary Fig. 1.** NMR measurement of ARS.

## Data Availability

All data is available upon request to the corresponding author.

## Abbreviations

PDT: Photodynamic therapy
MB: Methylene blue
ARS: Acetyl resistant starch
NPs: Nanoparticles
TEM: Transmission electron microscopy
DLS: Dynamic light scattering
CD: Circular dichroism
DMSO: Dimethyl sulfoxide
DMAP: Dimethyl aminopyridine
DCM: Dichloromethane
PVA: Poly (vinyl alcohol)
WAXRD: Wide-angle X-ray diffraction
SAXS: Small-angle X-ray spectrometer
DPBF: 1,4-Diphenyl-2,3-benzofuran
ROS: Reactive oxygen species
DCFH-DA: Dichlorofluorescein diacetate
