# Supplementary figures and images for: In vitro photodynamic therapy of methylene blue-loaded acetyl resistant starch nanoparticles
Source: Biomater Res. 2022 Jun 27;26:28. doi: 10.1186/s40824-022-00273-7 (PMC9235160; doi:10.1186/s40824-022-00273-7)

## Slide 1
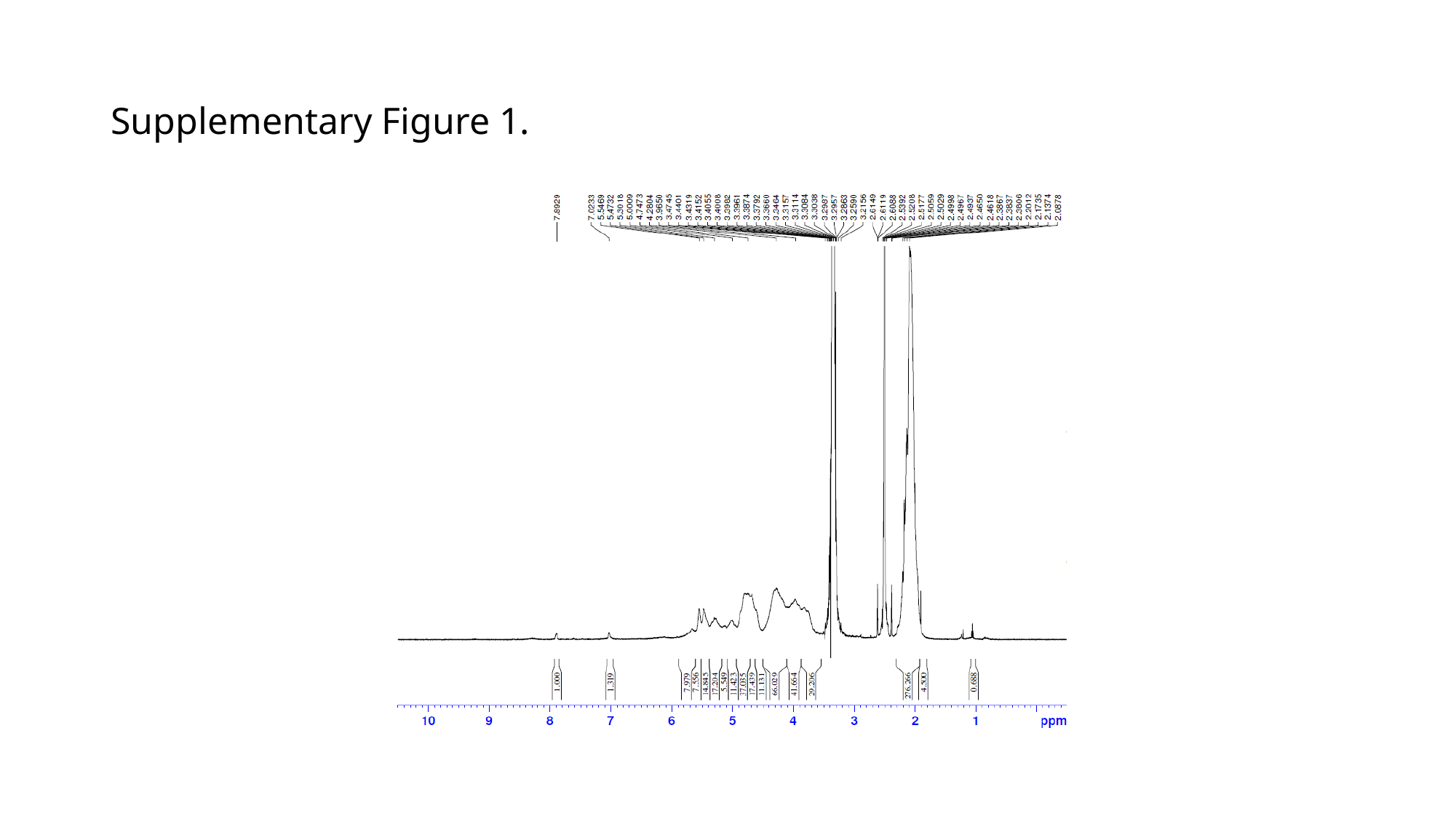

# Supplementary Figure 1.

Supplement: Supplementary file 1 — Additional file 1: Supplementary Fig. 1. NMR measurement of ARS. [file 40824_2022_273_MOESM1_ESM.pptx]
